# A computational study of action planning in syntax evolution

**DOI:** 10.1186/1471-2202-12-S1-P130

**Published:** 2011-07-18

**Authors:** Richard E Greenblatt

**Affiliations:** 1 Computational Sciences Research Center, San Diego State University, San Diego CA 92182, USA

## 

When we think about what makes humans distinct from other species, the use of language and the use of tools typically come to mind. However, we lack an adequate theory to account for the evolutionary origin and material substrate of these abilities [1]. In particular, we lack a bridging theory, that is, an explanatory framework that relates phenomena at one level (cognition) with another level (the brain). I hypothesize (a) that action planning [2] may form a key bridge linking linguistic and non-linguistic cognition; (b) that tool use and linguistic abilities may have co-evolved from simpler motor cognition; and (c) that this co-evolution may be understood in terms of the increasing topological complexity of brain network dynamical systems over evolutionary time. In order to test these hypotheses for plausibility, I have been developing a simple computer model which implements some of these ideas in the context of a highly idealized predator-prey interaction.

The computational model consists of two non-linguistic cognitive agents, herbivore ***H*** and predator ***P***, interacting with one another and with an environment ***E***. Both ***H*** and ***P*** move through ***E***, selecting and elaborating actions based on their goals and the perception of their environment. ***H*** has two goals: to find food and to avoid ***P***. ***P*** has only one goal: to catch ***H***. ***E*** an unbounded 2D plane, with a sparse set of points that have special properties, either as food or shelter points. Agents move through he environment according to a specified set of rules.

A basic assumption of the model is the necessity to account for the entire cognitive context, not simply the action planning proto-grammar. Each agent has a cognitive structure that is represented as a graph. Each node of the cognitive graph represents an hypothesized neurocognitive subsystem, implemented as a finite state machine. Each node is itself an autonomous agent, interacting with a subset of other nodes.

The nodes are connected by edges, through which information may be exchanged between connected nodes (generally bidirectional). The outermost node set is the sensorimotor layer. On the sensory side, the orientation and distance of environmental landmarks (food, shelter, other agents) are represented with respect to the current position of the agent. On the motor side, the currently invoked motor program is represented as the orientation, speed, and stopping condition of the current move. The representation layer associates properties (such as food or shelter) or actions (*e.g.*, move towards a target) with the sensorimotor field. The representation layer embodies action planning, combining sensorimotor representations with goals, as determined by the executive and integration layers. There is also an emotion layer, which assigns approach/avoidance valences to target locations.

The states of the action planning node implement a simplified proto-grammar, represented as a graph. The action representation graph has terminal nodes representing actions and their objects (or targets). The action representation dynamics are biased by inputs from the integration layer. The proto-grammar consists of two sorts of tokens (actions and objects) along with two operations (select and join). The selection operator selects an action, dependent on the states of other nodes. The join operator associates the selected action with a suitable object. The join operator therefore may be seen as an evolutionary precursor to the postulated linguistic merge operator [3]. Unlike merge, however, join is not recursive, in the current implementation of the model. In addition, operations that are conventionally segregated into semantic, pragmatic, and syntactic functions are mingled in the proto-grammar. In its present form, the model accounts only for productive (as opposed to receptive) aspects of action planning.

The model is still in an early of development. Results will be presented that correlate the internal model states (including sensitivity to the choice of model parameters) with the overt behavior of the agents, visualized as trajectories in the simulated 2D environment.
